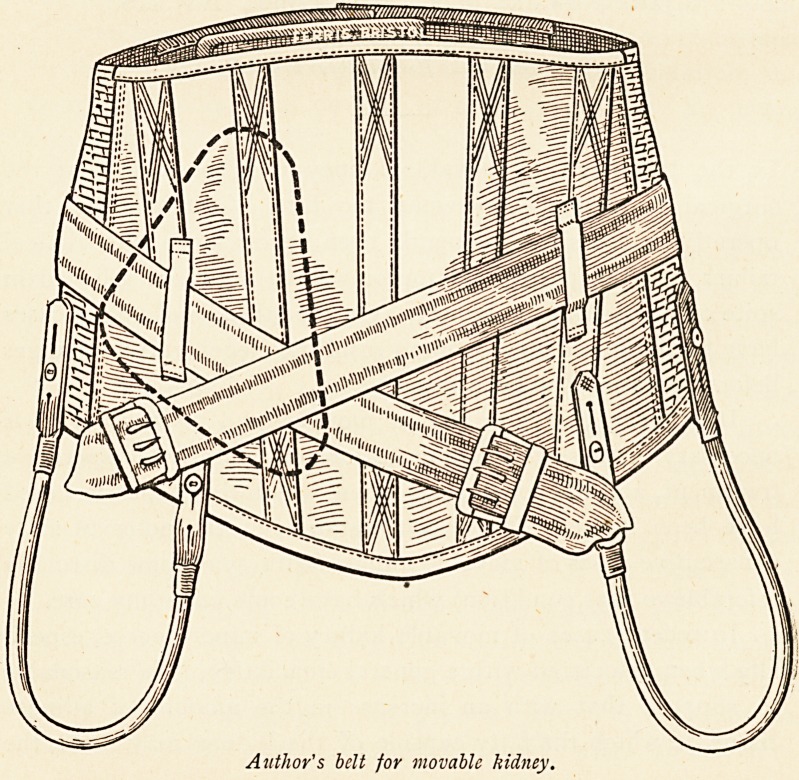# The Indications for Treatment in Nephroptosis

**Published:** 1902-12

**Authors:** James Swain

**Affiliations:** Professor of Surgery at University College, Bristol; Surgeon to the Bristol Royal Infirmary


### THE INDICATIONS FOR TREATMENT IN NEPHROPTOSIS.

BY

JAMES SWAIN, M.S., M.D. Lond., F.R.C.S.,

*Professor of Surgery at University College, Bristol;*
*Surgeon to the Bristol Royal Infirmary.*

IN the treatment of movable kidney it is possible that the surgical pendulum has swung too far. Certain it is that operative success has frequently been synonymous with clinical failure, and the patient's symptoms have not been relieved in spite of fixation of the kidney. On the other hand, many cases have been allowed to go on to resulting degenerative changes before operation has been resorted to.

It is clear, therefore, that a more careful discrimination is necessary in the selection of cases for non-operative or operative treatment, and the conclusions upon which this paper is based have been formed after a consideration of the notes of sixty consecutive cases of movable kidney (with symptoms distinctly referable to that condition) which have come under my care.

In recent cases of movable kidney of minor degree, especially when associated with a general emaciation, it is reasonable to suppose that with an increase in the amount of adipose tissue, in which the fatty capsule of the kidney may share, the organ may become fixed under suitable conditions of rest in the dorsal position, a liberal diet of fat-forming foods, and massage to prevent wasting of the muscles from disuse. It appears to be true that a movable kidney is spontaneously curable in these circumstances.

Most cases, however, require treatment by abdominal support or by operation.

### ABDOMINAL SUPPORT.

Some form of belt is all that is needed in many cases where the displacement does not exceed two or three inches; but where the range of movement is great and associated with "gastric crises" or symptoms of torsion of the renal vessels, abdominal supports often fail to give the necessary relief.

In cases complicated by hydronephrosis—for reasons which will appear later—belts are inadmissible.

*Author's belt for movable kidney.*

In all cases of movable kidney associated with hysterical and neurasthenic symptoms, or occurring as part of a general splanchnoptosis, belts should certainly be tried, and in many such cases they constitute the only justifiable form of treatment.

In younger women with strong abdominal muscles, belts are far more likely to fail than in older women who have borne many children, and whose abdominal walls are lax.

The beneficial influence of a belt is probably due to its support of the viscera in the lower abdomen, by which the kidney is indirectly prevented from moving from its accustomed site; but I am of opinion that a pad under the belt on the side of the abdomen affected is of accessory service, in spite of all that has been said to the contrary.

The abandonment of the pad by some surgeons, is largely due to the mechanical difficulty of exercising a sufficient direct pressure upon the pad without its becoming irksome to the patient. This difficulty can be overcome by a device which I have used for years with satisfaction, and which consists of a freely-running adjustable band passing obliquely round the outside of the belt guided by loops. This passes round the back, and both ends cross over the position of the junction of the middle and lower thirds of the pad placed under the belt, to be fastened by means of buckles securely fixed—one on either side—to the bottom of the belt. By drawing on the ends of the band when passed through the buckles, a direct pressure upwards and backwards is exerted upon the pad beneath.

Before the belt is applied it is of great importance that the movable kidney should be returned to its normal position.

### OPERATION (NEPHROPEXY).

The need for operation is the urgency of the case. When belts fail operative measures are indicated, and it has been already mentioned that a belt is often found to be inefficient in freely mobile kidneys associated with symptoms of gastric crises, or of torsion of the renal vessels. Under such circumstances operation may be undertaken with every hope of a satisfactory result.

The association of hydronephrosis may be regarded as imperatively necessitating surgical interference. Hydronephrosis implies kidney degeneration, and the condition tends to get worse the longer operation is delayed. In one of my cases pyelitis had actually been induced before the patient sought relief.

Such lesions constitute a menace to the life of the patient, and the earlier they are treated the better. Moreover, as is well known, the presence of degenerative changes in the kidneys renders operation more dangerous. In the only two deaths occurring in this series after nephropexy, both cases were associated with hydronephrosis.

Prevention is better than cure, and such considerations as these add greatly to the desirability of operative fixation in all cases of the more severe forms of renal mobility; and the small mortality attending the operation of nephropexy before degenerative changes have ensued, is more than balanced by the positive danger which exists in those cases to which reference has just been made.

In general terms it may be stated that nephropexy is more likely to relive painful symptoms than nervous phenomena, and the indications for operation are certainly weakest in cases of renal mobility associated with neurasthenia or hysterical symptoms, or when forming a part of a general splanchnoptosis. It is amongst such conditions that most of the cases will be found where operation has failed to relieve the patient of the more distressing symptoms; and in most of them general treatment, combined with the use of a belt, is all that is desirable.

In certain of these cases where the symptoms seem more especially referable to the kidney, it may occasionally be justifiable to resort to nephropexy in the hope of relieving the patients of the chief part of their sufferings; but failures are frequent, and before operation is undertaken the uncertainty of the result should be fully indicated.

It should be borne in mind that a large number of cases of movable kidney are unassociated with any subjective symptoms whatever, and because a movable kidney is found in a case presenting various hysterical phenomena it by no means follows that the former is the cause of the latter.

So too, when a movable kidney is only part of a general splanchnoptosis, it is not reasonable to suppose that fixation of the kidney will cure the patient of all the symptoms which such a case presents.

It is in cases of movable kidney in association with displacement of other organs that neurasthenia and hysterical symptoms are common, and then there is a double cause of a possible failure of the cure of the symptoms by operative procedure.

In the present series I have had seven cases of movable kidney in which displacements of the uterus also existed—in two of these hysteropexy was performed in addition to nephropexy—and others in association with prolapse of the ovaries or gastroptosis, and I have found treatment by nephropexy on the whole to be unsatisfactory.

Movable kidney should not be overlooked as a possible cause of intractable indigestion and other abdominal troubles, for which no sufficient reason can be given. Operation is necessary in many cases, but those who would avoid disappointment must exercise a wise judgment in the selection of cases for surgical treatment.

## Figures and Tables

**Figure f1:**